# *Alternaria* Species Associated with Strawberry Cultivars in the Almaty Region of Kazakhstan: Diversity, Multilocus Characterization and Pathogenicity

**DOI:** 10.3390/biology15201803

**Published:** 2026-10-09

**Authors:** Darya Bogatko, Rabiga Uakhit, Ainura Smagulova, Yuliya Yefremova, Vladimir Kiyan

**Affiliations:** 1 Laboratory of Plant Bioengineering, M.A. Aitkhozhin Institute of Molecular Biology and Biochemistry, Almaty 050012, Kazakhstan; bogatko@biocenter.kz (D.B.); uakhit@biocenter.kz (R.U.); smagulova@biocenter.kz (A.S.); yefremova@dku.kz (Y.Y.); 2 Laboratory of Biodiversity and Genetic Resources, National Center for Biotechnology, Astana 010000, Kazakhstan; 3 Educational Institution, Kazakh-German University in Almaty, Almaty 050010, Kazakhstan

**Keywords:** *Alternaria alternata*, *Alternaria tenuissima*, strawberry, pathogenicity, molecular identification, phylogenetic analysis, Kazakhstan

## Abstract

*Alternaria* species are important fungal pathogens of strawberry, while information on their occurrence and genetic diversity in Kazakhstan remains limited. This study aimed to characterize *Alternaria* isolates associated with symptomatic strawberry plants in the Almaty Region, to describe their recovery patterns among the examined cultivars and plant organs, and to assess the pathogenicity of representative isolates. Seventy plants representing nine strawberry cultivars were collected from four commercial farms. Fungal isolates were characterized based on cultural and morphological features and multilocus sequence analysis of the *ITS*, *GAPDH*, and *TEF1* regions, followed by maximum-likelihood phylogenetic analysis. *Alternaria* spp. were detected in 14 of 70 plants (20.0%), from which 19 isolates were obtained and molecularly characterized. Of these, 14 isolates (73.7%) were assigned to *A. alternata*, whereas five (26.3%) were assigned to *A. tenuissima*. The isolates were recovered from leaves, berries, stems, and roots and were detected in five of the nine cultivars examined. Multilocus phylogenetic analysis supported the placement of the *A. alternata* isolates within the *A. alternata* species complex, whereas the *A. tenuissima*-assigned isolates did not form a single exclusive lineage. A preliminary pathogenicity assay using two representative isolates showed that both were capable of inducing necrotic lesions and other disease symptoms on strawberry plants under controlled conditions; however, the limited number of isolates tested restricts inference to the broader recovered population. These findings provide new data on *Alternaria* associated with strawberry in the Almaty Region of Kazakhstan and highlight the value of multilocus approaches for their accurate characterization.

## Simple Summary

Strawberry plants can be affected by fungal diseases that reduce plant health and fruit production. Fungi of the genus *Alternaria* are important pathogens, but their diversity and effects on strawberries in Kazakhstan are poorly understood. We examined 70 plants representing nine cultivars from four commercial farms in the Almaty Region. *Alternaria* fungi were found in 14 plants, and 19 fungal samples were characterized using visible features and genetic information. Most were identified as *Alternaria alternata*, while others were closely related to *Alternaria tenuissima*. The fungi occurred on leaves, berries, stems, and roots. Two representative samples caused disease symptoms in preliminary tests. These findings provide useful information for monitoring strawberry diseases and improving crop protection in Kazakhstan.

## 1. Introduction

Strawberry (*Fragaria* × *ananassa Duch*.) is a high-value berry crop valued for its sensory and nutritional qualities; however, its productivity and marketability are strongly constrained by fungal and oomycete diseases [1]. Pathogens can infect all plant organs, causing losses both in the field and after harvest. *Botrytis cinerea*, the causal agent of grey mould, infects flowers and fruits and may remain quiescent until ripening, making it a major cause of postharvest rejection [2]. Other important diseases include anthracnose caused by *Colletotrichum* spp., powdery mildew caused by *Podosphaera aphanis*, leaf spots and blights, and crown and root rots associated with *Fusarium*, *Phytophthora*, *Neopestalotiopsis*, and related genera [3,4]. Disease development is promoted by susceptible cultivars, infected planting material, dense canopies, prolonged leaf wetness, and high humidity. Because symptoms caused by different fungi frequently overlap, visual diagnosis alone may lead to incorrect pathogen identification and inappropriate disease-management decisions.

Species of *Alternaria* are widespread saprotrophs, endophytes, and plant pathogens that can colonize leaves, stems, fruits, and senescent tissues [5,6]. On strawberries, *Alternaria* infection is commonly associated with brown to black necrotic spots, sometimes surrounded by chlorotic margins; lesions can expand and coalesce, reducing photosynthetic leaf area and fruit quality [7,8]. Some pathogenic lineages also produce host-selective or non-host-selective metabolites that contribute to tissue damage and disease development [9]. Reliable identification within *Alternaria* section *Alternaria* remains difficult because colony colour, conidial dimensions, septation, and sporulation patterns are variable and often overlap among closely related species. In particular, the distinction between *A. alternata* and *A. tenuissima* remains debated, while internal transcribed spacer (*ITS*) sequences alone generally provide insufficient resolution. Morphological assessment combined with multiple informative loci is therefore preferable, although species assignments should remain cautious when lineages are weakly resolved [10,11].

Complementary molecular approaches have been used to identify and characterize *Alternaria alternata*. In a study of pecan-associated isolates from South Africa, phylogenetic analysis combining *GAPDH*, *RPB2*, *TEF1*, and *Alt a1* sequences supported their identification as *A. alternata* sensu stricto [12]. PCR–restriction fragment length polymorphism (PCR-RFLP) analysis of the *Alt a1* region using HaeIII and HinfI differentiated restriction profiles among 222 isolates, providing a screening approach for detecting genetic variation [5]. Random amplified microsatellite (RAMS) analysis of 364 isolates further characterized genetic diversity and population structure, with most variation occurring within populations [6]. These studies illustrate the complementary roles of multilocus phylogeny and molecular fingerprinting in taxonomic identification and intraspecific characterization. However, variation detected by fingerprinting methods should not itself be interpreted as evidence of distinct species.

The *ITS* region, *GAPDH*, and *TEF1* were selected because they have been used in published multilocus studies of *Alternaria* and allow comparison with established reference strains. *ITS* provides a widely used fungal barcode, whereas the protein-coding loci *GAPDH* and *TEF1* provide additional sequence information for evaluating phylogenetic relationships [11]. However, their discriminatory power is limited within the section *Alternaria*. Several morphospecies, including *A. tenuissima*, could not be distinguished using their multilocus framework and treated them as synonyms of *A. alternata* [11]. Accordingly, the *ITS*–*GAPDH*–*TEF1* dataset was used to assess the phylogenetic affiliations of our isolates without assuming that it could definitively distinguish *A. alternata* from historically designated *A. tenuissima*. Assignments to the latter group are therefore considered provisional.

Research in countries neighbouring Kazakhstan demonstrates the regional importance of strawberry fungal diseases. In Beijing, China, Fu et al. recovered 102 *Alternaria* isolates from symptomatic strawberry leaves and reported *A. tenuissima* and *A. alternata* as the principal fungi associated with black spot [7]. A subsequent survey in Dandong examined 108 isolates and likewise detected both groups, with *A. tenuissima* predominating; pathogenicity was confirmed under controlled conditions [8]. In Western Siberia, Russia, common leaf spot, leaf scorch, and *Phomopsis* leaf blight caused by *Ramularia tulasnei, Marssonina potentillae*, and *Dendrophoma obscurans*, respectively, were identified as prevalent strawberry diseases [13]. These studies show that the composition of fungal pathogens varies by production region and must be determined locally.

Information from Kazakhstan remains limited. Preharvest grey mould caused by *B. cinerea* was reported from strawberry fields in the Semey region [14]. More recently, phytosanitary surveys in southeastern Kazakhstan detected *Fusarium* sp., *B. cinerea*, *Alternaria* sp., and *Rhizopus* sp.; however, multilocus data did not resolve the *Alternaria* isolates to species or lineage level, and pathogenicity was not evaluated [15]. Therefore, this study aimed to characterize *Alternaria* isolates associated with symptomatic strawberry plants from commercial farms in the Almaty Region using cultural and morphological characteristics and concatenated *ITS*, *GAPDH*, and *TEF1* sequences, to evaluate their provisional taxonomic assignments within *Alternaria* section *Alternaria*, to describe their recovery patterns among the sampled cultivars and plant organs, and to assess the pathogenicity of two selected isolates under controlled conditions.

## 2. Materials and Methods

The general study design included strawberry sampling, fungal isolation from various plant tissues, morphological and molecular identification of isolates, identification of *Alternaria* spp. species, pathogenicity testing, and data analysis.

### 2.1. Field Research

**Figure 1:**
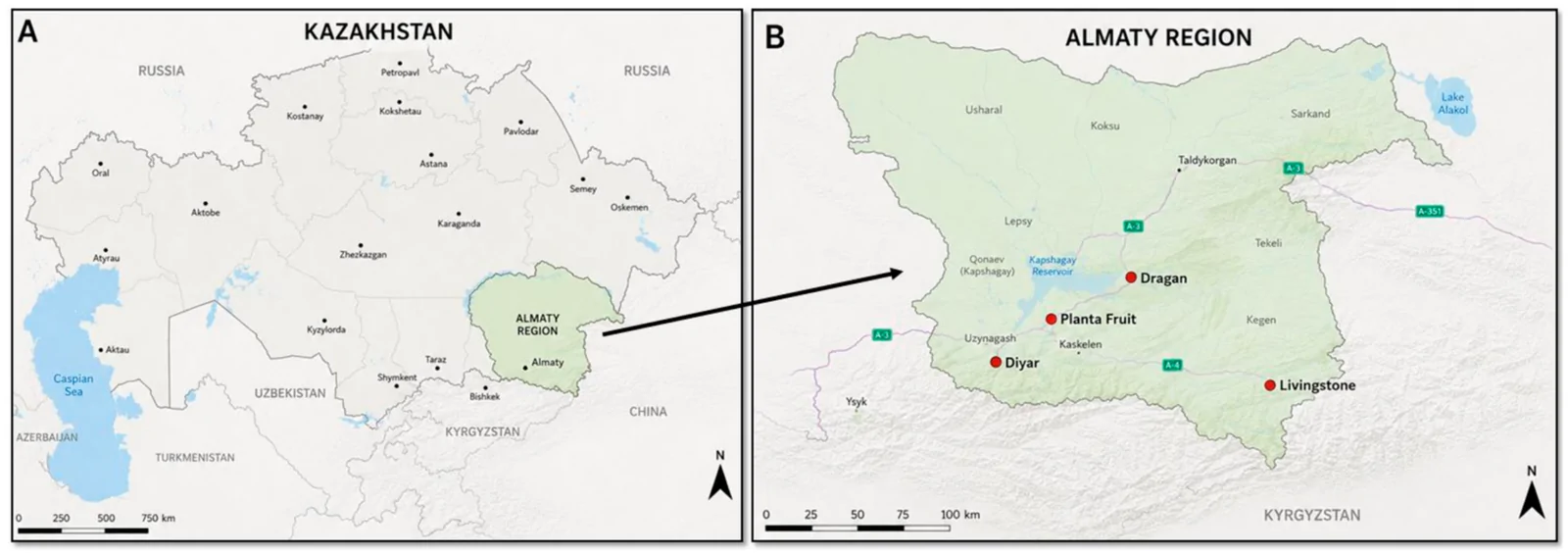
Map of collection points: (**A**) Map of Kazakhstan with the Almaty region highlighted in green; (**B**) enlarged map of the Almaty region showing the four strawberry farms where samples were collected.

Field research and sampling were carried out during the growing season across four farms in the Almaty region (Figure 1). Each farm was visited once during July–August 2025. Sampling was symptom-directed. Whole strawberry plants displaying visible disease symptoms were selected from different locations within each field, including peripheral and central areas, and transported to the laboratory for further examination.

A total of nine varieties were sampled from the farms: Vivara (n = 6), Murano (n = 10), Albion (n = 9), Clery (n = 17), Brilla (n = 4), Aprica (n = 4), Alba (n = 6), Florida (n = 4), and Asia (n = 10). Plants displaying a range of disease symptoms were selected from each variety, resulting in the collection of 70 plant samples.

Figure 1 shows the geographic distribution of the four strawberry farms surveyed in the Almaty Region, Kazakhstan. The sampling sites were located at the following geographic coordinates: Site 1, 43.359277° N, 77.268996° E; Site 2, 43.359810° N, 77.424620° E; Site 3, 43.362100° N, 77.270044° E; and Site 4, 43.202819° N, 76.570103° E. These sites represent the main commercial strawberry-growing areas included in the study.

### 2.2. Isolation and Cultivation of Pathogens

Plant material was collected from strawberry plants (*Fragaria* × *ananassa Duch*.) for the isolation of associated fungal pathogens. Leaves, stems, roots, and berries (if available) were taken from each plant. Tissue fragments were mainly selected from areas with visually pronounced lesion symptoms. Additionally, in some cases, visually asymptomatic leaves were selected from the same symptomatic plants. Such tissues were not considered as independent samples from asymptomatic plants. Prior to isolation, the plant material was thoroughly washed under running tap water to remove soil and other surface debris. Tissue fragments were then surface-sterilized with 70% ethanol for 5 min, followed by thorough rinsing with distilled water to remove residual ethanol. The sterilized fragments were dried on autoclaved filter paper for 5 min [16]. Small tissue fragments were subsequently excised from the samples and aseptically placed onto potato dextrose agar (PDA; Laboratorios Conda S.A., Torrejón de Ardoz, Spain).

The inoculated Petri dishes were incubated at 27 °C for 7–14 days. Fungal growth was monitored throughout the incubation period. Macromorphological characteristics of the developing colonies, including colony shape, colour, surface texture and growth rate, were recorded on days 5, 7, and 10 of incubation. Emerging fungal colonies were subcultured onto fresh PDA to obtain pure cultures. Since several types of tissues and tissue fragments of one plant were cultivated separately, several fungal cultures could be obtained from one plant. Each purified mushroom culture having cultural and morphological characteristics corresponding to *Alternaria* was accounted for as a separate pre-identified isolate. At the plant level, a plant was considered positive for *Alternaria* if at least one such crop was obtained from it.

The morphological characteristics of the fungal isolates were examined using a light microscope (AxioLab.A, Carl Zeiss, Göttingen, Germany) at 40×–100× magnification. Temporary microscopic preparations were prepared in glycerol and examined for the morphological features of fungal structures. Twenty conidia and twenty conidiophores from each morphological group were measured using light microscopy, and colony diameters were recorded on days 3 and 7 of cultivation. Preliminary identification of *Alternaria* isolates was performed based on their cultural and morphological characteristics using the identification manuals [17,18].

### 2.3. DNA Isolation

Genomic DNA was extracted from the fungal isolates using a modified CTAB method [19]. CTAB (≥99% purity; Carl Roth GmbH + Co. KG, Karlsruhe, Germany) was used for DNA extraction. Approximately 0.5–1 mg of fresh mycelial biomass grown on potato dextrose agar (PDA) was transferred to sterile 1.5 mL microcentrifuge tubes containing 500 μL of TES extraction buffer (100 mM Tris-HCl, pH 8.0; 10 mM EDTA; 2% SDS). The mycelial material was mechanically homogenized until a homogeneous suspension was obtained. Then, 5 µL of recombinant Proteinase K (PCR grade, ≥600 U/mL, approximately 20 mg/mL; Thermo Scientific, Waltham, MA, USA) was added to each sample, and the homogenates were incubated at 45 °C for 14–16 h. Following incubation, 100 μL of 5 M NaCl and 65 μL of 10% CTAB solution prepared in high-salt buffer and preheated to 65 °C were added to each sample. The samples were mixed gently and incubated at 65 °C for 10 min. For removal of proteins and other cellular components, an equal volume of chloroform-isoamyl alcohol (24:1, *v*/*v*) was added to each sample. The samples were gently mixed and centrifuged at 7000× *g* for 10 min. The upper aqueous phase was carefully transferred to a new sterile microcentrifuge tube. DNA was precipitated by adding 380 μL of chilled isopropanol, followed by incubation at −20 °C for 40 min [19]. The samples were then centrifuged at 12,000× *g* for 5 min, and the resulting DNA pellets were washed with 70% ethanol. Following an additional centrifugation step, the pellets were air-dried at room temperature until the residual ethanol had completely evaporated. The purified DNA was resuspended in 50 μL of TE buffer and stored at −20 °C until further molecular analysis. DNA concentration and purity were assessed using a NanoDrop 2000 spectrophotometer (Thermo Fisher Scientific, Waltham, MA, USA) by measuring the absorbance ratios at 260/280 and 260/230 nm.

### 2.4. PCR Amplification and Molecular Identification

For molecular identification and phylogenetic characterization of the fungal isolates, partial sequences of three genetic loci were amplified: the nuclear ribosomal internal transcribed spacer (*ITS*) region, comprising *ITS1*, the 5.8S rRNA gene, and *ITS2*; the glyceraldehyde-3-phosphate dehydrogenase (*GPDH*) gene; and the translation elongation factor 1-alpha (*TEF1*) gene. PCR amplification was performed using a SimpliAmp Thermal Cycler (Applied Biosystems, Foster City, CA, USA). PCR reactions were performed in a final volume of 25 μL. Each reaction contained 10 mMTris-HCl (pH 9.0), 50 mM KCl, 2.5 mM MgCl_2_, 0.2 mM of each dNTP, 1 μM of each primer, Taq DNA polymerase (Thermo Fisher Scientific, Vilnius, Lithuania), and 20–30 ng of template DNA. The reaction volume was adjusted to 25 μL with nuclease-free water. The primer pairs, target loci, and expected amplicon sizes are presented in Table 1.

For amplification of the *ITS* region, the PCR conditions consisted of an initial denaturation at 95 °C for 5 min, followed by 35 cycles of denaturation at 95 °C for 30 s, primer annealing at 55 °C for 30 s, and extension at 72 °C for 1 min. A final extension was performed at 72 °C for 7 min [20]. For amplification of the *GPDH* gene fragment, the PCR conditions consisted of an initial denaturation at 94 °C for 3 min, followed by 35 cycles of denaturation at 94 °C for 30 s, primer annealing at 59.5 °C for 30 s, and extension at 72 °C for 30 s. A final extension was performed at 72 °C for 10 min [21]. For amplification of the *TEF1* gene fragment, the PCR conditions consisted of an initial denaturation at 94 °C for 5 min, followed by 35 cycles of denaturation at 94 °C for 30 s, primer annealing at 55 °C for 40 s, and extension at 72 °C for 50 s. A final extension was performed at 72 °C for 7 min [22].

PCR products were separated by horizontal electrophoresis on 1% agarose gels prepared in 1× TAE buffer. A 100–1000 bp DNA ladder was used as a molecular size marker. Electrophoresis was performed at 120 V for 40 min, and the gels were visualized using SYBR™ Green I (Thermo Fisher Scientific, Eugene, OR, USA) and a GelDoc XR gel documentation system (Bio-Rad Laboratories, Hercules, CA, USA). Amplicons showing a single band of the expected size were selected for purification and subsequent sequencing. PCR products selected for sequencing were enzymatically purified using Exonuclease I (New England Biolabs (NEB), Ipswich, MA, USA). Each PCR product was treated with Exonuclease I and incubated at 37 °C for 30 min, followed by enzyme inactivation at 85 °C for 15 min, according to the manufacturer’s instructions.

Purified amplicons were sequenced using the Sanger sequencing method with the corresponding forward and reverse amplification primers and the BigDye Terminator v3.1 Cycle Sequencing Kit (Applied Biosystems, Foster City, CA, USA). Sequencing reactions were analyzed using a 3730xl DNA Analyzer with a 96-capillary array (Applied Biosystems, Thermo Fisher Scientific, Foster, CA, USA). The resulting nucleotide sequences were deposited in the GenBank database and compared with reference sequences using BLASTn; accession numbers and reference sequences are provided in Table 2. The BLAST matches used to support preliminary taxonomic assignments showed 98–100% sequence identity and 95–100% query coverage. The identified sequences were subsequently used for phylogenetic analysis.

### 2.5. Phylogenetic Analysis

Sequence ends were examined and trimmed in BioEdit version 7.0. Terminal regions containing insertions, deletions, or mismatches considered indicative of low-quality sequencing were removed following manual inspection. The internal transcribed spacer region of ribosomal DNA (*ITS*; amplified using the *ITS1/ITS4* primer pair), partial glyceraldehyde-3-phosphate dehydrogenase gene (*GAPDH*), and partial translation elongation factor 1-alpha gene (*TEF1*) sequences were aligned separately using CLUSTALW in MEGA11. The alignments were manually inspected in BioEdit to identify apparent alignment errors and discrepancies, subsequently concatenated in MEGA11, and exported in FASTA format. The final concatenated dataset contained 1370 nucleotide positions and comprised 31 sequences, including 19 isolates obtained in the present study, reference sequences of Alternaria spp. retrieved from GenBank, and the outgroup. Table 2 provides each study isolate’s code and the corresponding GenBank accession numbers for *ITS*, *GAPDH*, and *TEF1*.

The concatenated *ITS–GAPDH–TEF1* dataset was analyzed as a single, unpartitioned alignment using the maximum-likelihood method in MEGA11 with the Tamura–Nei nucleotide substitution model specified a priori. Formal model selection and locus-specific partitioning were not performed; applying a single substitution model across the three loci does not account for possible differences in their evolutionary patterns and represents a limitation of the analysis. Branch support was assessed using 1000 bootstrap replicates.

Phylogenetic relationships were inferred using the maximum-likelihood method implemented in MEGA version 11 and the Tamura–Nei nucleotide substitution model [23,24]. Initial trees for the heuristic search were generated automatically using the Neighbor-Joining and BioNJ algorithms based on a matrix of pairwise distances calculated according to the Tamura–Nei model. The topology with the highest log-likelihood value was selected. First-, second-, and third-codon positions, as well as noncoding positions, were included. The tree was drawn to scale, with branch lengths representing the number of substitutions per site. *Stemphylium vesicarium* was used as the outgroup to root the tree. The resulting tree had a log-like lihood value of −3767.79.

### 2.6. Assessing the Pathogenicity of Fungal Isolates

The pathogenicity of the isolated fungal strains was evaluated by artificially inoculating strawberry plants, followed by confirmation of Koch’s postulates [25,26]. Two representative isolates were selected for the pathogenicity test: *A. alternata* 1.1.71A, originally isolated from the stem of the Vivara variety at Farm1, and *A. tenuissima* 1.2.129, originally isolated from the root of the Murano variety at Farm1. These isolates were selected because they were among the first pure cultures and had already been confirmed in the process of molecular identification, representing two groups of *Alternaria* obtained during the study. Prior to the experiment, the isolates were cultured on potato dextrose agar (PDA) at 25 °C for 10–12 days. To prepare the inoculum, conidia were washed from the agar surface using sterile saline solution [26,27]. The final conidial concentration used for inoculation was 2 × 10^6^ conidia/mL for *A. alternata* isolate 1.1.71A and 1 × 10^7^ conidia/mL for *A. tenuissima* isolate 1.2.129.

To assess the pathogenicity of the isolated *A. alternata* and *A. tenuissima* strains, eight healthy strawberry seedlings were used. The plants were approximately 1.5–2 months old; prior to inoculation, they were kept individually in plastic containers under greenhouse conditions. The plants belonged to the same varieties from which the isolates were isolated. Before inoculation, the leaves were not mechanically damaged. For each fungal isolate, three plants were artificially inoculated, while one additional plant served as the corresponding uninoculated control. Thus, each isolate was tested on three experimental plants with a separate control plant. A volume of 100–200 µL of the prepared conidial suspension from the corresponding isolate was applied to the leaf blades of the experimental plants. Leaf inoculation was chosen as a standardized model for assessing pathogenicity, since leaf symptoms were among the most commonly observed manifestations of the disease during plant examinations and allowed for a reproducible visual assessment of symptom development. The experiment was aimed at evaluating the pathogenic potential of the isolates on strawberry plants, rather than at reproducing the original organ-specific infection pathway associated with the tissue from which each isolate was isolated. To maintain high humidity, the inoculation sites were covered with sterile, moistened cotton wool and aluminum foil for 48 h. Control plants received an equivalent volume of sterile saline solution instead of the conidial suspension.

The plants were incubated at 25 ± 2 °C with 70–90% relative humidity and a 12 h/12 h light/dark photoperiod. Disease development was assessed qualitatively by visual observation. The plants were monitored for the presence of necrotic or dark spots on the leaves and stems, chlorosis, deformation and curling of the leaf blades, as well as wilting. A quantitative disease severity index was not calculated. Fungi were re-isolated from symptomatic tissues on PDA medium, and the morphological characteristics of the resulting cultures were compared with those of the corresponding original isolates. This procedure was used to assess the experimental re-isolation criterion and to support the pathogenic potential of the tested isolates [25,26]. Since the inoculum concentrations for each isolate are different, the pathogenicity test was interpreted as qualitative and was not used for statistical or quantitative comparison of the virulence or aggressiveness of the two isolates.

## 3. Results

### 3.1. Symptoms Observed on Strawberry Plants

**Figure 2:**
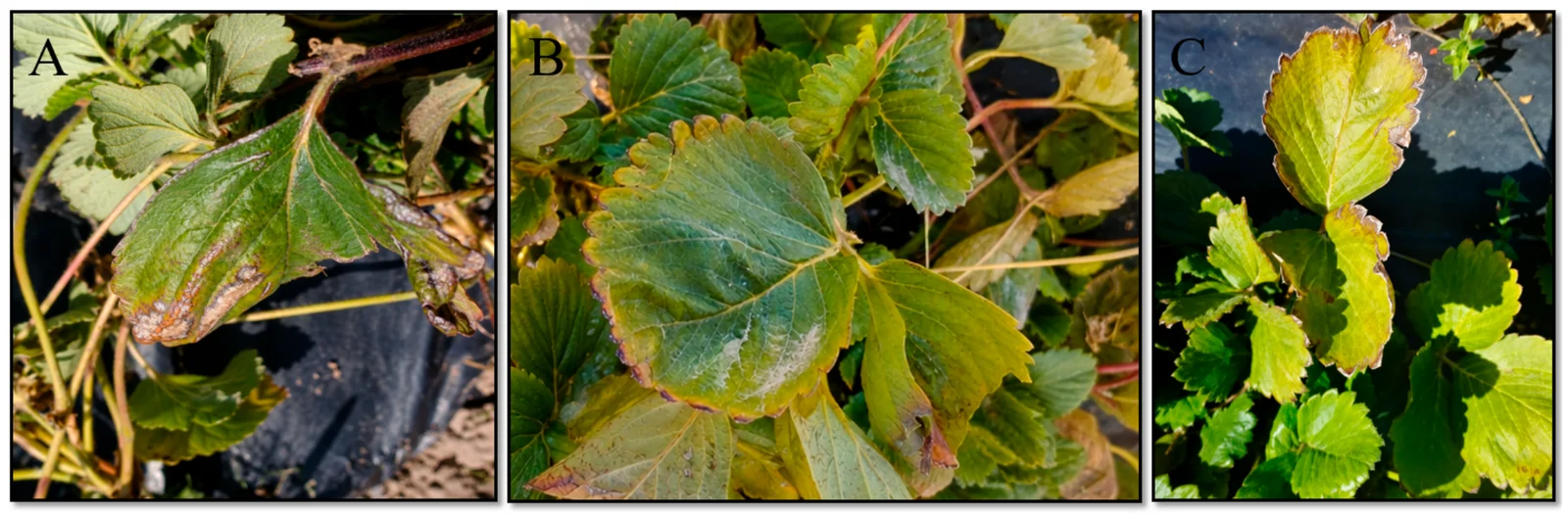
Field symptoms observed on strawberry plants: (**A**) leaf with marginal necrosis, tissue browning, and deformation; (**B**) leaf showing marginal necrosis accompanied by yellowing and drying of the affected tissue; (**C**) severe leaf damage characterized by extensive marginal necrosis, drying, and deformation of the leaf blade. The presented symptoms reflect field observations and should not be interpreted as being caused exclusively by *Alternaria* spp.

A total of 70 strawberry plants representing nine cultivars were collected from four commercial farms in the Almaty Region. During the phytosanitary survey, plants exhibited a range of foliar symptoms, including marginal necrosis, tissue browning, chlorosis, drying, deformation, and curling of the leaf blades (Figure 2).

The most prominent symptoms were necrotic lesions occurring along the margins of the leaf blades (Figure 2A–C). Affected tissues became brown, dry, and brittle, with a distinct yellowish or light-green transition zone between healthy and necrotic tissues. Necrosis occurred either continuously along the leaf margins or as discrete lesions. In severely affected leaves, necrotic areas extended over a substantial portion of the leaf margin and were accompanied by drying and deformation of the distal parts of the leaf blade, whereas the central portions often remained green. Some affected leaves exhibited wrinkling and slight inward curling toward the center of the rosette (Figure 2A,B).

**Figure 3:**
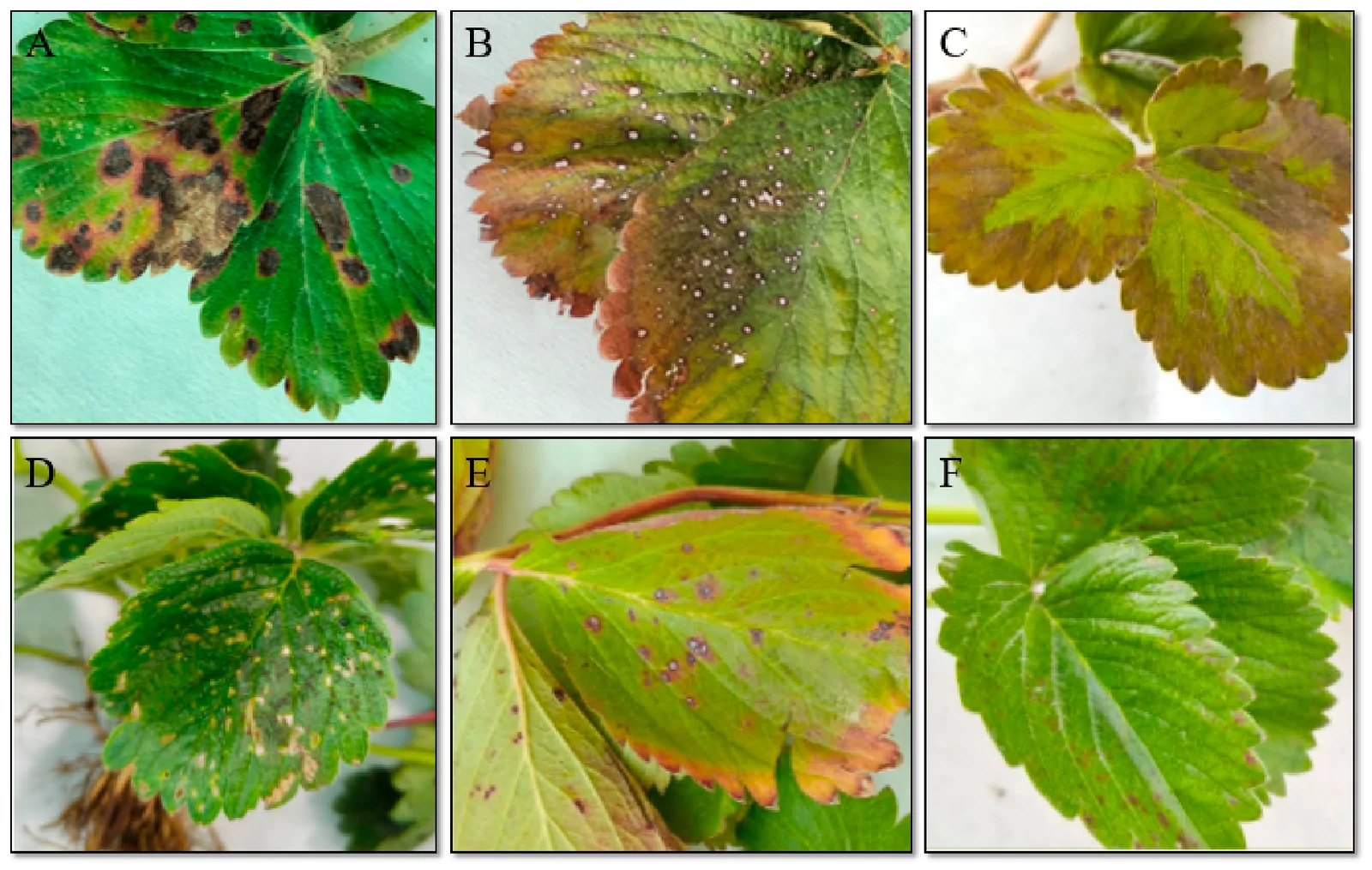
Different types of leaf damage observed in strawberry plants: (**A**) irregularly shaped necrotic lesions on the leaf blade; (**B**) extensive necrotic areas with browning and drying of the affected tissues; (**C**) marginal necrosis of the leaf blade; (**D**) necrotic lesions accompanied by yellowing of the surrounding tissues; (**E**) multiple small necrotic lesions distributed across the leaf surface; (**F**) leaf without visible symptoms. The presented symptoms reflect field observations and should not be interpreted as being caused exclusively by *Alternaria* spp.

In addition to marginal necrosis, leaves with lesions of different shapes, sizes, and degrees of severity were observed (Figure 3). These included round or irregularly shaped necrotic spots (Figure 3A), extensive necrotic areas associated with browning and drying, and multiple small lesions distributed across the leaf surface (Figure 3B,D). In some cases, yellowing was observed around the necrotic areas (Figure 3C,E). Individual lesions occasionally enlarged and coalesced, resulting in larger areas of necrotic tissue. Apparently symptom-free leaves from the same symptomatic plants were also sampled and used for fungal isolation (Figure 3F).

### 3.2. Morphological Characteristics of the Isolated Cultures of Alternaria spp.

As a result of isolation and subsequent subculturing, 70 pure fungal cultures were obtained and preliminarily assigned to the genus *Alternaria* based on their cultural and morphological characteristics. All isolates demonstrated stable growth on the culture medium and retained their characteristic features during repeated cultivation.

**Figure 4:**
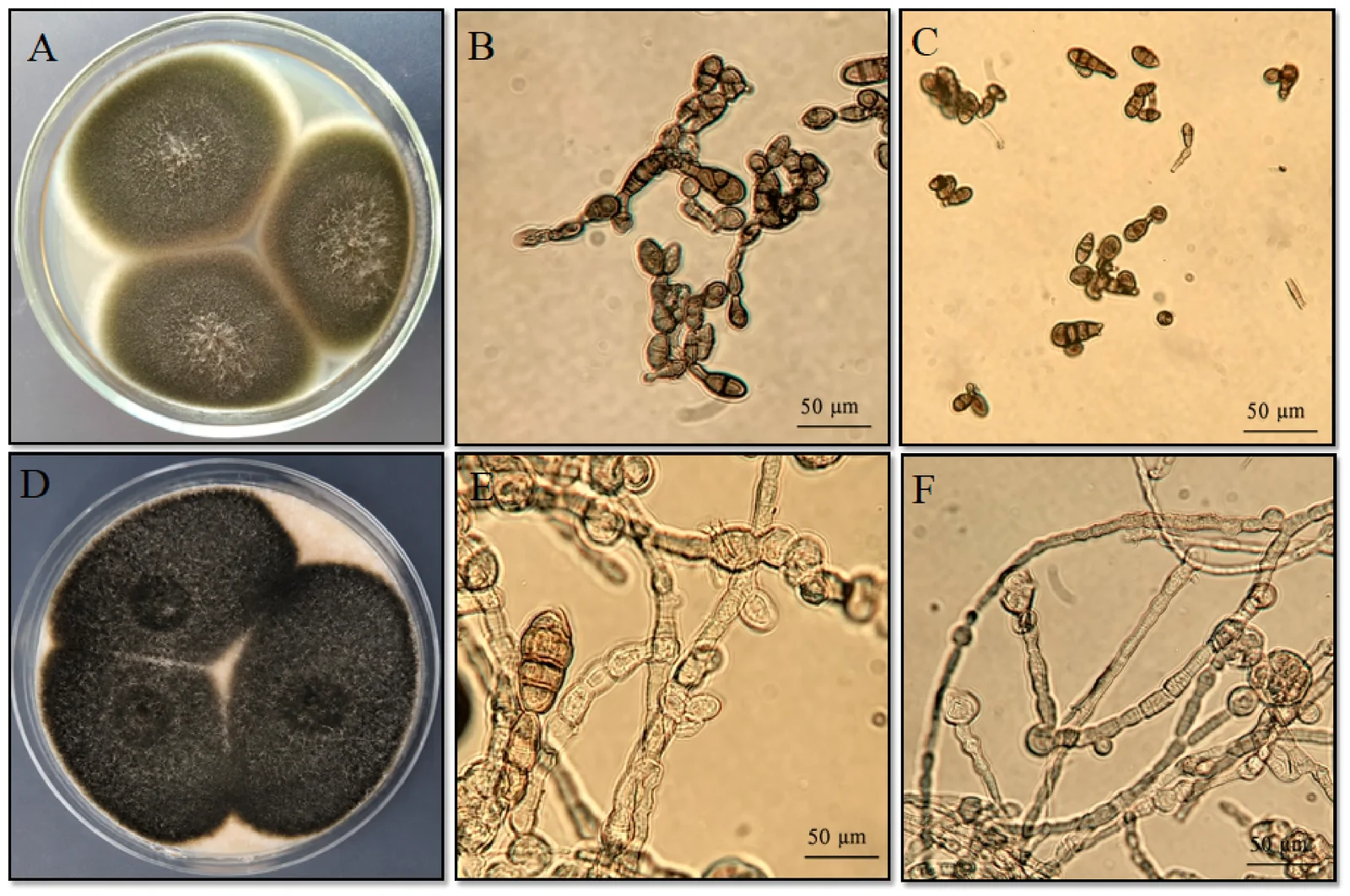
Cultural and morphological characteristics of representative *Alternaria* isolatesselected after multilocus identification: (**A**) colony of *A. alternata* on culture medium; (**B**) mycelium of *A. alternata*; (**C**) conidia of *A. alternata*; (**D**) colony of *A. tenuissima* on culture medium; (**E**) mycelium of *A. tenuissima*; (**F**) conidia of *A. tenuissima*.

During the early stages of cultivation, the colonies developed light-colored mycelium, which gradually became gray, olive-gray, or dark brown. Most isolates produced dense aerial mycelium with a distinct radial growth pattern. Variation among isolates was observed in the intensity of sporulation and development of aerial mycelium. At later stages of cultivation, colony pigmentation increased, with the central part of most colonies becoming dark olive to almost black, while the peripheral areas remained lighter. Colony margins were predominantly smooth or slightly wavy (Figure 4A,D). Quantitative measurements of colony diameter showed that, on the third day of cultivation, colonies assigned to *A. alternata* reached approximately 32–34 mm in diameter, whereas colonies assigned to *A. tenuissima* reached 34–37 mm. By the seventh day, colony diameters increased to 57–60 mm for *A. alternata* and 62–65 mm for *A. tenuissima*. Overall, after 7 days of incubation, the colony diameter of the examined *Alternaria* cultures ranged approximately from 55 to 65 mm.

For morphological illustration, two representative isolates were selected following multilocus identification, one from each of the two *Alternaria* groups identified.

Microscopic examination revealed dark, septate mycelium and *Alternaria* conidia that were predominantly club-shaped or ellipsoidal, with transverse and longitudinal septa (Figure 4B,C,E,F). Morphometric measurements were taken on 20 conidia per morphological group. Conidia of *A. alternata* measured 18.4–40.6 × 8.1–9.5 µm, where as those of *A. tenuissima* measured 16.7–42.8 × 7.0–14.1 µm. In both groups, conidia contained 1–4 transverse septa and 0–2 longitudinal septa. Conidia were produced in branched and unbranched chains; chain length generally ranged from 3 to 5 conidia in *A. alternata* and from 6 to 15 in *A. tenuissima*.

Conidiophore morphology was also assessed quantitatively. Measurements were based on 20 conidiophores for each morphological group. Conidiophores were relatively short in both groups, measuring 21.4–55.6 × 3.0–5.3 µm in *A. alternata* and 22.8–62.3 × 3.1–5.0 µm in *A. tenuissima*. Overall, the observed morphological characteristics were consistent with those of the genus *Alternaria*. The two morphological groups differed in colony diameter, conidial dimensions, conidiophore length, and conidial chain length, whereas septation patterns largely overlapped. Among the isolates, differences were observed in growth rate, sporulation intensity, colony pigmentation, and overall colony morphology. Because closely related *Alternaria* taxa may share overlapping morphological characteristics, the quantitative morphological data were used as supporting phenotypic evidence rather than as the sole basis for species-level identification. These characteristics enabled the preliminary assignment of the isolates to the genus *Alternaria*. Because closely related *Alternaria* species may exhibit overlapping morphological characteristics, species-level identification was subsequently performed using multilocus molecular analysis and sequencing of the corresponding amplicons.

### 3.3. Occurrence and Recovery Patterns of Alternaria spp. in the Examined Strawberry Samples

Because sampling was symptom-directed and the numbers of examined plants varied among cultivars and farms, the observed recovery frequencies are presented descriptively and should not be interpreted as estimates of disease prevalence or as direct comparisons of cultivar susceptibility.

Of the 70 strawberry plants examined, *Alternaria* spp. were isolated from 14 plants, representing 20.0% of the symptom-directed sample (Table 3). This figure reflects the proportion of *Alternaria*-positive plants and should not be interpreted as isolate prevalence. Within the examined samples, *Alternaria* spp. were recovered from 8 of 22 plants in Farm 1 (36.4%). Among individual cultivar–farm combinations, 3 of 4 Albion plants examined in Farm 4 yielded *Alternaria* spp. (75.0%). No *Alternaria* spp. were isolated from the Asia, Alba, Brilla, or Florida cultivars.

A total of 70 pure cultures preliminarily assigned to *Alternaria* were recovered from the 14 *Alternaria*-positive plants based on cultural and morphological characteristics. Molecular identification using the *ITS*, *GPDH*, and *TEF1* loci yielded consistent sequence data for 19 isolates, which were subsequently used for species-level identification. The remaining cultures were excluded because amplification was unsuccessful for one or more loci, as indicated by the absence of amplicons of the expected size on agarose gel electrophoresis, or because the three loci produced inconsistent taxonomic assignments, including identification as other fungal species. The molecularly characterized isolates originated from leaves, berries, stems, and roots and were obtained from five of the nine cultivars examined (55.6%): Vivara, Murano, Albion, Clery, and Aprica.

The largest proportion of molecularly confirmed isolates was obtained from leaves (9 isolates, 47.4%), followed by berries (7, 36.8%), stems (2, 10.5%), and roots (1, 5.3%). Thus, the majority of isolates were recovered from above-ground plant organs.

The recovery of molecularly confirmed isolates varied among the examined farms. Farm 1 yielded 12 isolates (63.2%), followed by Farm 4 with 4 isolates (21.1%), Farm 3 with 2 isolates (10.5%), and Farm 2 with 1 isolate (5.3%) (Table 3). At the cultivar level, the largest number of isolates was obtained from Albion (6 isolates, 31.6%), followed by Vivara and Murano (5 isolates each, 26.3%), Aprica (2 isolates, 10.5%), and Clery (1 isolate, 5.3%).

Molecular identification showed that 14 of the 19 isolates (73.7%) were assigned to *A. alternata*, whereas 5 isolates (26.3%) were assigned to *A. tenuissima*. *A. alternata* was detected in the Vivara, Albion, Clery, and Aprica cultivars, whereas *A. tenuissima* was detected exclusively in Murano. Across farms, *A. alternata* was identified in Farms 1, 3, and 4, while *A. tenuissima* was detected in Farms 1 and 2. Regarding plant organs, *A. alternata* was mainly recovered from leaves and berries and was also detected in stems, whereas *A. tenuissima* was recovered from leaves, berries, stems, and roots.

### 3.4. Phylogenetic Analysis

**Figure 5:**
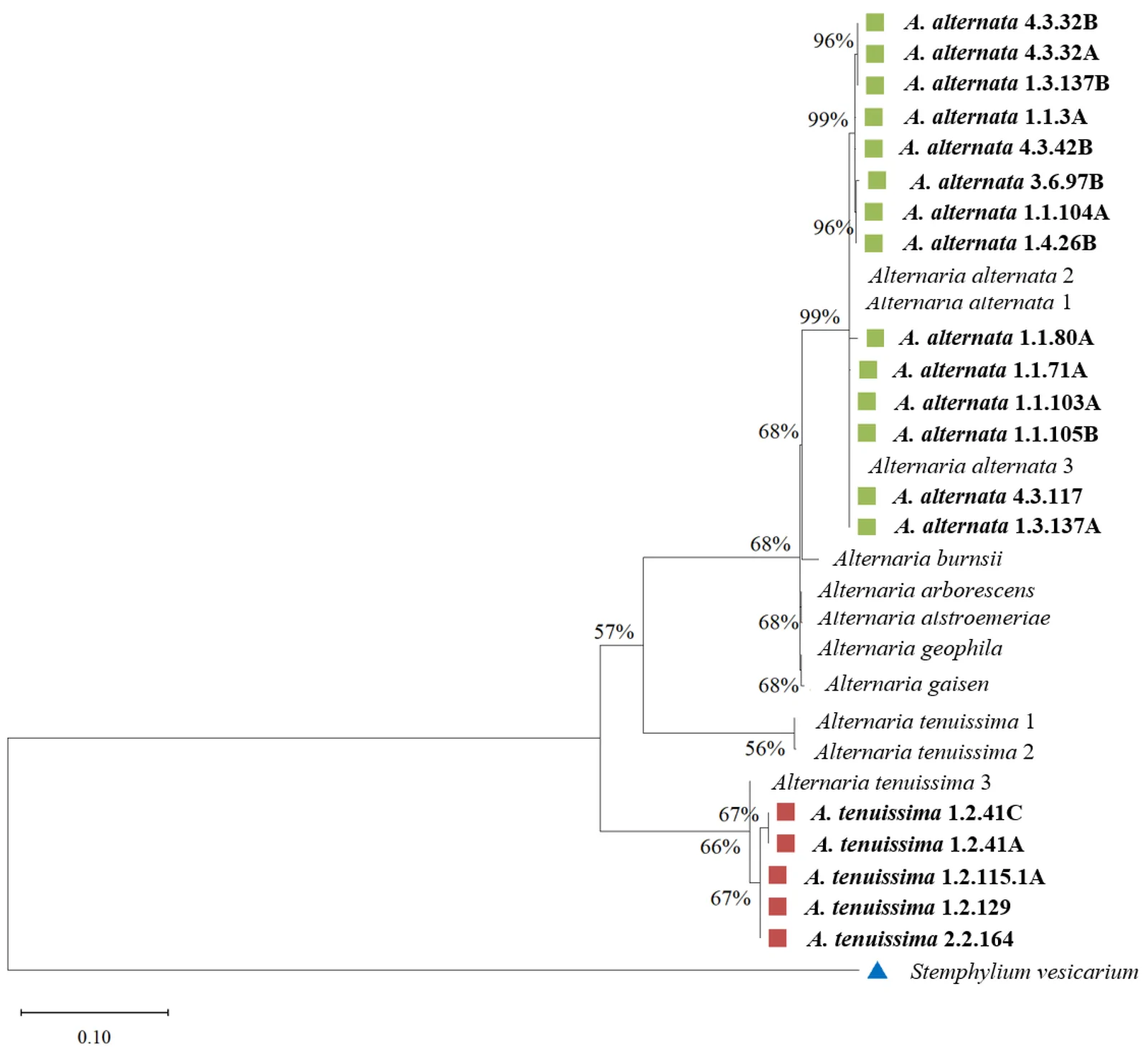
Maximum-likelihood phylogenetic tree of *Alternaria* spp. inferred from the concatenated *ITS*, *GAPDH*, and *TEF1* nucleotide sequence dataset using the Tamura–Nei model in MEGA11. Green squares indicate entries assigned to the *A. alternata* group, and red squares indicate entries assigned to the *A. tenuissima* group. Isolates obtained in this study are identified by bold labels containing their isolate codes; reference taxa are shown in regular type. Branch lengths represent substitutions per site. *Stemphylium vesicarium*, indicated by a blue triangle, was used as the outgroup. Bootstrap replicates: 1000.

The concatenated *ITS–GAPDH–TEF1* phylogeny was used to examine the phylogenetic affiliations of the study isolates within *Alternaria* section *Alternaria* (Figure 5). The isolates were provisionally assigned to *A. alternata*-associated and *A. tenuissima*-associated groups; these assignments do not represent conclusive species-level separation based on the three analyzed loci.

Fourteen isolates (4.3.32B, 4.3.32A, 1.3.137B, 1.1.3A, 4.3.42B, 3.6.97B, 1.1.104A, 1.4.26B, 1.1.80A, 1.1.71A, 1.1.103A, 1.1.105B, 4.3.117, and 1.3.137A) were provisionally assigned to the *A. alternata*-associated group. Their positions in the concatenated tree were interpreted alongside the included reference sequences, without treating this assignment as evidence of a distinct, conclusively resolved species lineage.

The five isolates provisionally assigned to the *A. tenuissima*-associated group (1.2.41C, 1.2.41A, 1.2.115.1A, 1.2.129, and 2.2.164) clustered with the reference sequence designated *A. tenuissima* 3. Isolates 1.2.41A and 1.2.41C formed a pair within this cluster. However, the reference sequences designated *A. tenuissima* 1 and *A. tenuissima* 2 occupied a separate lineage. Thus, the study isolates and reference sequences carrying the *A. tenuissima* designation did not collectively form a single monophyletic group, further limiting interpretation of this designation as a resolved species boundary.

The analysis also included reference sequences designated *A. burnsii, A. arborescens, A. alstroemeriae, A. geophila*, and *A. gaisen*. *Stemphylium vesicarium* was included as the designated outgroup. Overall, the phylogeny provided a framework for evaluating provisional affiliations rather than definitive discrimination between *A. alternata* and *A. tenuissima.*

### 3.5. Pathogenicity of A. alternata and A. tenuissima on Strawberry Plants

**Figure 6:**
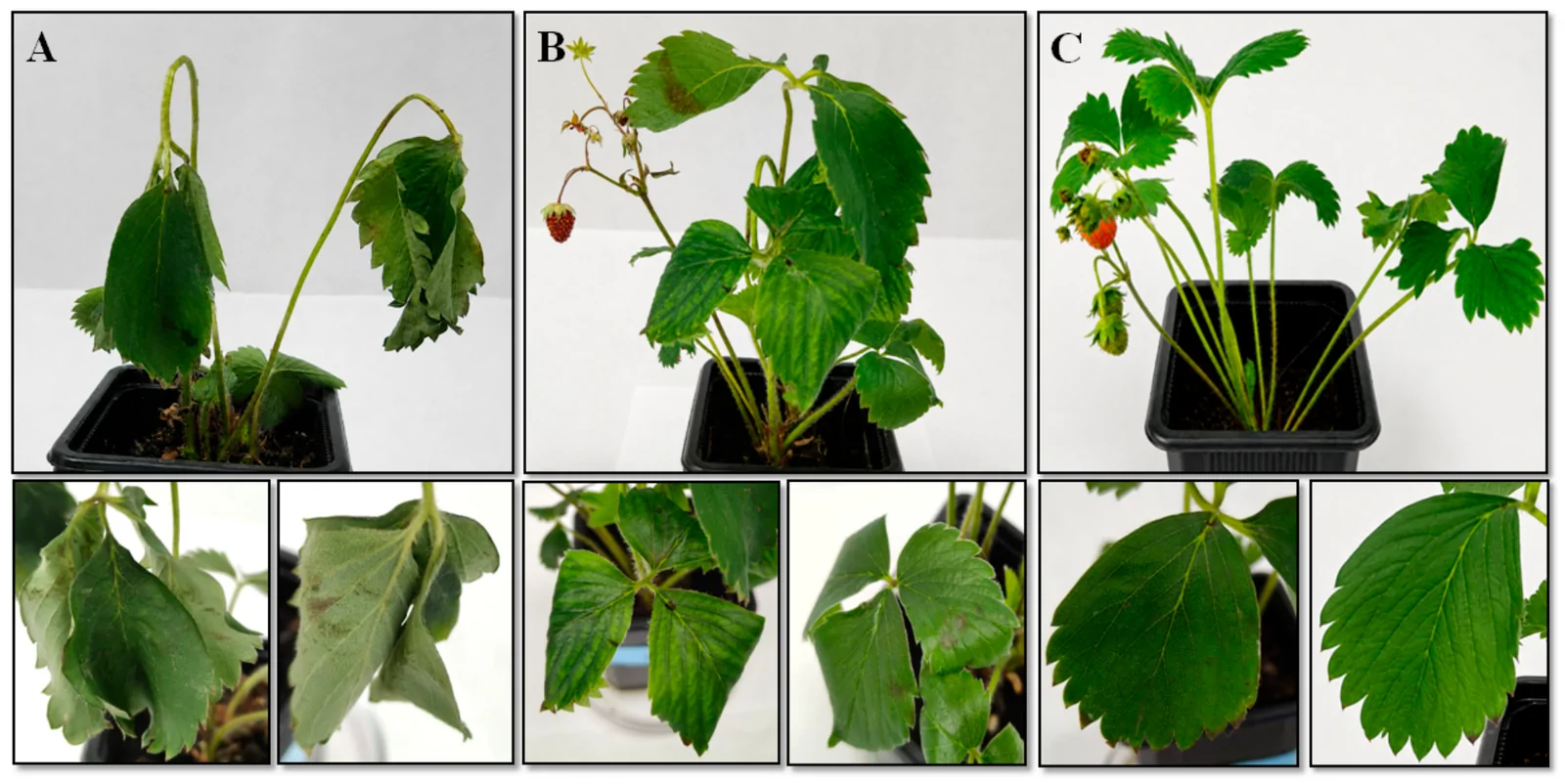
Results of the pathogenicity test for *A. alternata* and *A. tenuissima* on strawberry plants 10 days after inoculation: (**A**) strawberry plant inoculated with *A. tenuissima*; (**B**) strawberry plant inoculated with *A. alternata*, showing necrotic lesions, chlorosis, and deformation of the leaf blades; (**C**) uninoculated control plant without visible symptoms.

The pathogenicity of *A. tenuissima* and *A. alternata* was assessed on healthy strawberry plants under controlled conditions. The first symptoms appeared on the third day after inoculation and were characterized by small, dark lesions on the leaves. By the tenth day, symptoms had become more pronounced in plants inoculated with both Alternaria species, including leaf curling, dark necrotic lesions on the leaves, and dark lesions on the stems (Figure 6A,B). In contrast, the control plants showed no visible symptoms (Figure 6C).

*Alternaria* cultures were successfully re-isolated from symptomatic tissues of the inoculated plants. The morphological characteristics of the re-isolated cultures matched those of the corresponding original isolates. No *Alternaria* isolates were recovered from the control plants. These observations supported the pathogenic potential of both isolates tested. The pathogenicity assay was interpreted qualitatively, and no statistical comparison of pathogenicity, virulence, or aggressiveness was made between the two isolates.

## 4. Discussion

The cultural and microscopic characteristics of the *Alternaria* isolates obtained in the present study were generally consistent with those previously described for *Alternaria* associated with strawberry. The isolates showed variation in colony pigmentation and sporulation, while septate mycelia and conidia with transverse and longitudinal septa were consistently observed. Sun et al. [8] reported similar characteristicsfor *A. alternata* and *A. tenuissima* isolated from strawberry in Dandong, China. Takahashi et al. [28] likewise found no substantial differences in colony colour, conidial shape, size, or septation between strawberry-pathotype isolates originating from Japan and New Zealand. These findings emphasize the usefulness of morphological characteristics for preliminary identification, while also demonstrating their limited discriminatory value among closely related *Alternaria* spp.

In the present survey, *Alternaria* spp. were detected in 14 of 70 strawberry plants (20.0%). Among the 19 molecularly confirmed isolates, *A. alternata* predominated (73.7%), whereas *A. tenuissima* accounted for 26.3%. In contrast, Fu et al. [7] reported that *A. tenuissima* represented 59.8% and *A. alternata* 40.2% of 102 isolates obtained from strawberry in Beijing. Similarly, Sun et al. [8] found a predominance of *A. tenuissima* (72.2%) over *A. alternata* (27.8%) among 108 isolates from Dandong. Such differences may reflect geographic variation in *Alternaria* populations, cultivar composition, environmental conditions, and sampling strategies. Because these studies differed in sampling design and reporting units, the observed proportions of *A. alternata* and *A. tenuissima* should be compared primarily in terms of species composition rather than directly as measures of prevalence.

Most isolates in our study originated from leaves (47.4%) and berries (36.8%), whereas stems (10.5%) and roots (5.3%) were less frequently represented. The predominance of leaf-derived isolates agrees with studies from Beijing and Dandong, where *A. alternata* and *A. tenuissima* were frequently recovered from symptomatic strawberry leaves [7,8]. The recovery of isolates from berries also supports previous reports of *Alternaria* associated with strawberry fruit rot [29].

Differences were additionally observed among cultivars: *Alternaria* was detected in Vivara, Murano, Albion, Clery, and Aprica but not in Asia, Alba, Brilla, or Florida. Cultivar-dependent responses to *Alternaria* have previously been demonstrated experimentally. Takahashi et al. [30] found only ‘Morioka-16’ and ‘Robinson’ to be susceptible among 60 tested strawberry cultivars and breeding lines, while a subsequent study demonstrated that susceptibility to the strawberry pathotype was genetically determined [28]. However, because cultivar representation and growing conditions differed among farms in our survey, the observed distribution should not be interpreted as direct evidence of cultivar resistance or susceptibility.

The concatenated analysis of *ITS*, *GAPDH* and *TEF1* dataset provided greater phylogenetic information than would generally be expected from the *ITS* region alone. Similar three-gene datasets have previously been used for maximum-likelihood identification and placement of *Alternaria* species [31,32]. The placement of all 14 numbered *A. alternata* isolates together with the corresponding reference sequences was consistent with their identification as members of the *A. alternata* species complex. Their short branch lengths also indicated high sequence similarity and limited variation across the examined loci.

The five isolates identified as *A. tenuissima* formed a recognizable group with one *A*. *tenuissima* reference sequence. Nevertheless, the separation of the other two *A. tenuissima* references demonstrates that the name *A. tenuissima* did not correspond to a single exclusive lineage in the present analysis. Thus, the tree should not be interpreted as conclusive evidence that *A. alternata* and *A. tenuissima* represent two fully independent phylogenetic species.

This result agrees with the recognized difficulty of distinguishing small-spored species within *Alternaria* section *Alternaria*. Woudenberg et al. [11,33] found that numerous traditionally recognized morphospecies could not be reliably separated even using seven loci and genome-level comparisons. Under this taxonomic framework, *A. tenuissima* has been treated as a synonym of *A. alternata*. Therefore, the isolates labelled *A. tenuissima* in the present study may represent a genetically differentiated lineage or morphotype within *A. alternata* sensu lato rather than an independently resolved species [34]. Accordingly, the species names used here are retained to describe the molecularly supported clustering of the isolates, but their taxonomic status should be interpreted with caution.

The present study also expands the currently limited molecular information on *Alternaria* associated with cultivated strawberry in Kazakhstan and provides baseline data for the Almaty Region. The detection of two closely related *Alternaria* spp. across multiple farms, cultivars, and plant organs indicates that the fungal diversity associated with strawberry in the region is not restricted to a single species. These findings provide a basis for further investigations of the geographic distribution, host association, and epidemiological significance of *Alternaria* in strawberry production systems in Kazakhstan.

The pathogenicity assessment demonstrated that representative isolates assigned to *A. alternata* and *A. tenuissima* were capable of inducing disease symptoms on healthy strawberry plants under controlled conditions. Initial symptoms appeared three days after inoculation as small dark lesions, while by day 10, more pronounced necrotic lesions, leaf curling, and dark lesions on stems were observed. *Alternaria* was subsequently re-isolated from symptomatic tissues, whereas the studied fungi were not recovered from control plants, supporting the pathogenic potential of the tested isolates and satisfying the experimental criteria of Koch’s postulates.

Similar responses have been reported in other studies of strawberry-associated *Alternaria*. Sun et al. [8] observed dark-brown lesions, leaf deformation, and yellow halos after inoculation of strawberry leaves with *A. alternata* and *A. tenuissima*, with *A. alternata* showing higher mean disease incidence and disease index values. Fu et al. [7] likewise demonstrated that isolates of both groups obtained from strawberry black spot were capable of inducing characteristic foliar symptoms. Pathogenic potential has also been demonstrated on strawberry fruits: Li et al. [29] reported disease development following inoculation with representative *A. alternata* and *A. tenuissima* isolates and subsequently re-isolated the fungi from symptomatic fruits. The agreement between these previous observations and the present pathogenicity assay supports the potential role of *Alternaria* in strawberry disease development, although differences in isolate origin, inoculum concentration, host material, and experimental conditions should be considered when comparing pathogenicity results among studies.

Taken together, the results demonstrate that *Alternaria* was recovered from a subset of the symptom-directed strawberry samples collected from the four surveyed farms in the Almaty Region. The predominance of *A. alternata*, together with the detection of *A. tenuissima*-assigned isolates and the demonstrated pathogenic potential of representative isolates, indicates that multiple members of *Alternaria* section *Alternaria* may contribute to disease symptoms in strawberry in the region. At the same time, the phylogenetic results highlight the taxonomic complexity of these closely related species and support the use of multilocus or genome-scale approaches for their accurate characterization.

Several limitations of the present study should be considered. First, sampling was restricted to four commercial farms within the Almaty Region, and the number of plants examined for individual cultivars and farms was not uniform, which limits the generalisability of the observed distribution patterns. Second, although the multilocus *ITS*–*GAPDH*–*TEF1* dataset provided useful information for species identification, resolution of closely related species within *Alternaria* section remains challenging, and the phylogenetic placement of the *A. tenuissima*-assigned isolates should therefore be interpreted with caution. Third, the pathogenicity assay was conducted using only two representative isolates and a limited number of plants; therefore, the results demonstrate pathogenic potential but do not allow comparison of virulence or aggressiveness among isolates or extrapolation to the entire population of *Alternaria* recovered in this study. Further studies involving broader geographic sampling, additional genomic markers or genome-scale data, and larger pathogenicity experiments will be required to clarify the diversity, host association, and pathogenic variation in *Alternaria* in strawberry in Kazakhstan.

## 5. Conclusions

This study provides the first molecularly characterized data on *Alternaria* associated with cultivated strawberry examined at four commercial farms in the Almaty Region of Kazakhstan. Isolates provisionally assigned to *A. alternata* constituted the majority of the molecularly characterized isolates, while *A. tenuissima*-assigned isolates were also detected across different cultivars and plant organs. Multilocus phylogenetic analysis supported the close relationship among the analyzed isolatesand highlighted the taxonomic complexity of the *Alternaria* section. Pathogenicity assays demonstrated the pathogenic potential of representative *A. alternata* and *A. tenuissima* isolates on strawberry plants. These findings mayprovide baseline information for further studies on the diversity, distribution, and pathogenic significance of *Alternaria* in strawberry production in Kazakhstan.

## Figures and Tables

**Table 1:** Primers used for identification of *Alternaria* spp.

Target	Name	Sequence (5′–3′)	Amplicon Size, bp	Reference
*ITS*	ITS-1 F	TCCGTAGGTGAACCTGCGG	520–550	White et al. [20]
	ITS-4 R	TCCTCCGCTTATTGATATGC		
*GPDH*	GPD-1 F	CAACGGCTTCGGTCGCATTG	450	Berbee et al. [21]
	GPD-2 R	GCCAAGCAGTTGGTTGTGC		
*TEF1-α*	TEF-1 F	CTTGGAGGGAACCATCTTGA	~600	Sommaetal. [22]
	TEF-2 R	CTGGTACAAGGGTTGGGAGA		

**Table 2:** GenBank accession numbers of *ITS*, *GPDH*, and *TEF1* sequences of *Alternaria* isolates obtained in this study and references used in the phylogenetic analysis.

Organism Name	Isolate	*ITS* Accession Number	*GPDH* Accession Number	*TEF1* Accession Number
The isolates obtained in this study				
*Alternaria alternata*	1.1.3A	PZ390046	PZ414629	PZ414641
*Alternaria alternata*	4.3.32B	PZ390047	PZ414630	PZ414642
*Alternaria alternata*	4.3.42B	PZ390048	PZ414631	PZ414643
*Alternaria alternata*	1.1.71A	PZ390049	PZ414632	PZ414644
*Alternaria alternata*	1.1.80A	PZ390050	PZ414633	PZ414645
*Alternaria alternata*	1.1.103A	PZ390051	PZ414634	PZ414646
*Alternaria alternata*	1.1.105B	PZ390052	PZ414635	PZ414647
*Alternaria tenuissima*	1.2.41C	PZ390053	PZ414636	PZ414648
*Alternaria alternata*	4.3.117	PZ390054	PZ414637	PZ414649
*Alternaria alternata*	1.3.137A	PZ390055	PZ414638	PZ414650
*Alternaria alternata*	1.3.137B	PZ390056	PZ414639	PZ414651
*Alternaria tenuissima*	2.2.164	PZ390057	PZ414640	PZ414652
*Alternaria tenuissima*	1.2.115.1A	PZ424329	PZ810351	PZ923799
*Alternaria tenuissima*	1.2.41A	PZ424330	PZ810352	PZ923800
*Alternaria tenuissima*	1.2.129	PZ424331	PZ810353	PZ923801
*Alternaria alternata*	4.3.32A	PZ424332	PZ810354	PZ923802
*Alternaria alternata*	1.4.26B	PZ424333	PZ810355	PZ923803
*Alternaria alternata*	1.1.104A	PZ424334	PZ810356	PZ923804
*Alternaria alternata*	3.6.97B	PZ424335	PZ810357	PZ923805
The reference sequences from GenBank				
*A. alternata 1*	CBS 916.96	AF347031	AY278808	KC584634
*A. alternata 2*	CBS 102604	KP124334	AY562410	KP125110
*A. alternata 3*	CBS 102596	KP124328	KP124183	KP125104
*A. tenuissima 1*	CBS 918.96	AF347032	AY278809	KC584693
*A. tenuissima 2*	CBS 880.95	KP124322	KP124177	KP125098
*A. tenuissima 3*	CBS 965.95	KP124323	KP124178	KP125099
*A. geophila*	CBS 101.13	KP124392	KP124244	KP125170
*A. aff. arborescens*	Not stated	KP124399	KP124251	KP125177
*A. gaisen*	CBS 632.93	KC584197	KC584116	KC584658
*A. burnsii*	CBS 107.38	KP124420	JQ646305	KP125198
*A. alstroemeriae*	CBS 118809	KP124297	KP124154	KP125072
*Stemphylium vesicarium (outgroup)*	CBS 191.86/E.G.S. 36-138.2	KC584239	AF443884	KC584731

**Table 3:** Distribution of *Alternaria* spp. samples among the studied strawberry varieties in various farms.

Farm	Variety	Plants Examined, n	Positive Plants, n	Plant Organ				Molecularly Confirmed Isolates, n	Positive Plants, %
				Root, n	Stem, n	Leaf, n	Berry, n		
1	Vivara	6	3	–	*A. alternata* (1)	*A. alternata* (3)	*A. alternata* (1)	5	50.0
	Murano	5	2	*A. tenuissima* (1)	–	*A. tenuissima* (1)	*A. tenuissima* (2)	4	40.0
	Clery	6	1	–	–	*A. alternata* (1)	–	1	16.7
	Albion	5	2	–	–	–	*A. alternata* (2)	2	40.0
Total		22	8	1	1	5	5	12	36.4
2	Murano	5	1	–	*A. tenuissima* (1)	–	–	1	20.0
Total		5	1	0	1	0	0	1	20.0
3	Asia	5	0	–	–	–	–	0	0
	Alba	6	0	–	–	–	–	0	0
	Aprica	4	2	–	–	–	*A. alternata* (2)	2	50.0
	Brilla	4	0	–	–	–	–	0	0
	Clery	7	0	–	–	–	–	0	0
		26	2	0	0	0	2	2	7.7
4	Asia	5	0	–	–	–	–	0	0
	Albion	4	3	–	–	*A. alternata* (4)	–	4	75.0
	Clery	4	0	–	–	–	–	0	0
	Florida	4	0	–	–	–	–	0	0
Total		17	3	0	0	4	0	4	17.6
Overall		70	14	1	2	9	7	19	20.0

## Data Availability

The original contributions presented in this study are included in the article. Further inquiries can be directed to the corresponding author.

## Abbreviations

The following abbreviations are used in this manuscript:

bp	base pairs
GAPDH	glyceraldehyde-3-phosphate dehydrogenase
ITS	internal transcribed spacer
ML	maximum likelihood
PDA	potato dextrose agar
TEF1	translation elongation factor 1-alpha
